# Understanding Socio‐Material Relations in Nurse Staffing Systems: Insights From a Qualitative Study in England and Wales

**DOI:** 10.1111/nin.70008

**Published:** 2025-03-11

**Authors:** Davina Allen, Heather Strange, Nina Jacob, Giulia Zoccatelli, Amit Desai, Anne Marie Rafferty

**Affiliations:** ^1^ School of Healthcare Science Cardiff University Cardiff UK; ^2^ Centre for Trials Research Cardiff University Cardiff UK; ^3^ Florence Nightingale School of Nursing and Midwifery, and Palliative Care King's College London London UK

**Keywords:** actor network theory, case studies, practice‐approach, qualitative research, safe nurse staffing systems, socio‐materiality, workforce

## Abstract

Amidst a global nursing shortage, ensuring sufficient nurses are available to care for patients is an international policy priority. High‐income countries have developed and implemented numerous models to ensure safe nurse staffing, yet evidence to recommend any single methodology remains limited. Existing research primarily evaluates nurse staffing systems by inputs and outcomes, neglecting their internal dynamics. Using qualitative case study data from England and Wales and drawing on practice perspectives and Actor Network Theory, this paper examines these socio‐material relations. Healthcare systems are complex, diverse and constantly evolving. Whilst identifying a single ‘best’ model may prove both impractical and elusive, this paper demonstrates the value of this theoretical approach for understanding the interplay of system components and the mediating effects of context. These insights can inform future research and help improve systems to meet the demands of late modernity.

## 1 Introduction

Over the last two decades, high‐income countries have developed a range of systems to ensure adequate numbers of nurses are available for patient care. These include strategies determined by individual health organisations, through policy‐led approaches, to models mandated in law. Diverse methodologies have emerged, including professional judgement approaches, which involve expert nurses extrapolating from shift planning to determine the staff to be employed on the unit; bench‐marking approaches, where staffing need is established by reference to comparator units; time‐task approaches, which involve assessment of the time associated with care plan activities; volume‐based approaches that prescribe nurse‐patient ratios or nursing care hours for specific clinical areas; patient classification approaches, in which nursing workload is assessed by categorising individual patients according to care needs; indicator approaches, where patient needs are assessed along a number of dimensions; and regression approaches, which model the relationship between patient, ward and organisational factors to obtain the recommended staffing establishment for a particular unit (Griffiths et al. 2020a). Despite considerable investment and activity, however, recent reviews (Van den Heede et al. 2020; Griffiths et al. 2020a; Twigg et al. 2021) have concluded there is not one best way to address this issue. Extensive evidence links lower nurse staffing levels to poorer patient outcomes and staff experience in acute care settings (Aiken et al. 2014) and in the context of a global nursing workforce crisis and widespread professional unrest (Buchan and Catton 2023), optimising nurse staffing systems has become a priority across international contexts. There is a substantial research literature on nurse staffing systems. Most studies can be characterised as black box evaluations (Lipsey 1993); they have typically focused on inputs and outcomes whilst leaving the inner workings of staffing systems hidden from view. But nurse staffing systems are complex interventions; they contain several interacting components – formal methods, measurement tools, technologies, policies, knowledge and organisational actors – which are conditioned by the contexts in which they are implemented (Skivington et al. 2021). In this paper, we deploy a practice approach and insights from Actor Network Theory (ANT) (Latour 2005) to look inside nurse staffing systems in England and Wales. As the first empirical study to adopt a socio‐material perspective, we demonstrate how this approach enhances understanding of the core components of nurse staffing systems, their interrelationships and mechanisms, and the mediating effects of context, providing insights for improvement and future research.

## 2 Materials and Methods

### 2.1 Research Aims

The primary aim of the study was to explore the role of nurses' professional judgement in nurse staffing systems. A secondary aim was to compare policy differences between England and Wales to augment understanding.

### 2.2 Policy Context

Nurse staffing became a priority in the UK following a public inquiry into poor care and elevated levels of patient mortality at Stafford Hospital (Francis 2013). Previously, decisions about nurse staffing and skill‐mix were determined by local organisations, but in the wake of the Francis Inquiry, national policies were developed. Health policy in the UK is devolved. In Wales, the responsibilities for nurse staffing in adult medical and surgical wards and children's wards are mandated by the Nursing Staffing Levels (Wales) Act 2016, whereas in England, nurse staffing follows policy guidance. England is often characterised as an outlier among high‐income countries in having a flexible and non‐prescriptive nurse staffing regime (Van den Heede et al. 2020). In practice, however, a de facto national system has emerged. Many of the ‘recommendations’ outlined in policy guidance are operationally mandatory because they are subject to external regulation by the Care Quality Commission which derives substantial powers of inspection and enforcement from statute (National Quality Board 2016; NHS Improvement 2018).

### 2.3 Study Design

The study had a descriptive qualitative cross‐case comparative design centred on adult in‐patient services in three University Health Boards in Wales (WCS1, WCS2, WCS3) and three NHS Trusts in England (ECS1, ECS2, ECS3) (self‐citation × 2). Adult in‐patient settings were selected because the statutory duties of the Wales Act were limited to these areas at the time of the study. Cases were chosen to represent a variety of district general and tertiary hospitals in urban, rural and city locations. The research team was independent of all cases.

### 2.4 Theoretical Framework

Data generation and analysis was informed by a practice approach (Nicolini 2012) and drew on insights from ANT. Founded on the shared orientations of praxeology (Bourdieu 1977), ethnomethodology (Garfinkel 1967), structuration (Giddens 1984) and activity theory (Engestrom 2000), a practice approach attends to how agents interact with the socio‐material conditions of the practice context in pursuit of their objectives. It has four theoretical premises. First, social phenomena are conceptualised as on‐going practical accomplishments produced through human agency. This premise directed attention to the processes and concerns through which safe staffing was negotiated by organisational actors. Second, practice theory emphasises that human activity is enabled by artefacts. This premise focused attention on the material (technologies, forms and tools) and psychological artefacts (concepts, categories, representations) deployed in nurse staffing systems. Third, practices are understood as emerging from the dynamic interaction of human actors with the material and social world as people find solutions to their problems. This premise directed attention to how nurse staffing systems were enacted within the organisational and material constraints of the case study contexts. Fourth, a practice approach underlines how relationships created in networks of practices can service certain interests (Ortner 1984). This premise directed attention to the distribution of power and privilege in nurse staffing systems and how this impacted decision‐making. Finally, from a practice perspective, knowledge is understood as the capacity to undertake a social and material activity. This premise directed attention to the systems of knowledge enrolled in nurse staffing systems and their interrelations.

ANT complements a practice approach because it affords an analytic sensitivity to the socio‐material *relationships* within a field. Characterised by its symmetrical treatment of people and inanimate actors, ANT attends to the division of activities between humans and non‐humans. Studying nurse staffing systems through the lens of ANT entailed careful analysis of these relationships. We analysed tools, technologies and artefacts, the activities that were ‘delegated’ to them, and the tasks they ‘prescribed’ to users. We attended to the ‘scripts’ (Akrich 1992) that were embedded in nurse staffing technologies, that is, the ways of seeing and acting they conveyed. We also focused on how agency was distributed between human and non‐humans and with what effects. Combining ANT with a practice‐based approach enabled us to trace the socio‐material relations in nurse staffing systems in each of the case studies, the mediating effects of context, and their implications for workforce planning, staff experiences and the quality of patient care.

### 2.5 Data Generation

Data were generated by H.S. and N.J. through stakeholder interviews (nonclinical senior nurses; executive board members; non‐nursing middle manager; practice educators; ward‐based nursing staff), observations of staffing meetings, acquisition of materials (policies, documents and artefacts), and focused observations in clinical areas (3 cases) (Table 1). In each case, stakeholders, materials and meetings were identified through snowball sampling. Whilst Covid restrictions limited access to clinical settings, we were able to observe staffing meetings which, as a result of the pandemic, were undertaken virtually rather than in‐person. Most meetings were recorded digitally; where digital recording was not possible, they were recorded as fieldnotes. Interviews took place using secure video‐conferencing software (Microsoft Teams), were guided by semi‐structured templates tailored to organisational roles and lasted between 20 min and an hour. Researchers had flexibility to probe areas of interest. Interviews were primarily designed to understand the practices involved in nurse staffing systems – how things were done, the tools that were used for this purpose, and the contribution of different actors – although respondents also offered their appraisals of the operation of nurse staffing systems. In addition, we invited participants to describe the pre‐pandemic operation of staffing systems, and to reflect on the impacts of Covid. Clinical observations in three cases were undertaken by shadowing senior nurses and ward managers and recorded as low‐inference fieldnotes, which documented observations and conversations as they happened without interpretation (see Table 1 for a data summary). Data were generated between January 2021 and March 2023. Ethics approval was granted by the Cardiff University School of Healthcare Sciences Research Ethics Committee. All participants gave written consent.

**Table 1:** Case study data summary.

Data sources	Case studies					
	WCS1	WCS2	WCS3	ECS1	ECS2	ECS3
Observations (clinical)	7 h	0	14 h	0	14 h	0
Observations (meetings)	4	1	4	3	3	13
Interviews: operational nursing staff (e.g. ward managers, senior/lead nurses, matrons)	3	1	8	4	8	4
Interviews: senior nursing staff (e.g. directors of nursing, workforce leads, chief nurses)	5	8	7	6	2	4
Interviews: non‐nursing senior and managerial staff (e.g. finance managers, HR managers, chief operating officers, medical directors)	1	3	0	1	1	9

### 2.6 Analysis

Transcribed fieldnotes, interviews and pertinent sections of staffing meetings were uploaded into qualitative data analysis software (NVivo11) and coded to facilitate data extraction and management. Documents, technologies and artefacts were analysed manually. These were treated as a resource to enable understanding of staffing systems and also analysed as actors in everyday practices.

Analysis proceeded as follows. First, we developed detailed theoretically informed empirical descriptions of the network of socio‐material relations comprising the nurse staffing systems in each case. Second, an additional coding frame was applied to the data to explore the role of professional judgement in nurse staffing systems (primary research aim). These findings are reported elsewhere (Allen et al. 2023). Third, we compared the wider operation of nurse staffing systems across cases (secondary research aim). This involved analysing policy documents through a socio‐material lens to develop a theoretically informed explanation of the core components of nurse staffing systems in England & Wales and undertaking a cross‐case comparative analysis of the operation of nurse staffing systems in the study site and the conditioning effects of context. These findings form the basis of this paper.

The emerging analysis was shared with case study representatives via two online workshops, one for Wales and one for England, with participant insights informing the final analysis. D.A. led the overall analysis, N.J. and H.S. were responsible for phases 1 and 2, and A.D. and G.Z. were responsible for phase 3. A.M.R. offered independent critical interpretation.

## 3 Findings

Despite devolution, we found considerable policy convergence between England and Wales. Both systems required healthcare organisations to implement ward‐to‐board responsibility for safe staffing, undertake formal biannual reviews of unit staffing establishments, and develop operational policies to mitigate risk when there was a discrepancy between capacity and demand. Both polices utilised ‘triangulated’ methodologies, in which quantitative data from workload measurement tools and Nurse Sensitive (quality) Indicators (NSI), are combined with ‘professional judgment’ for workforce planning. Overall, nurse staffing systems followed a common pattern.

Our analysis proceeds as follows. First, we describe the contextual features which conditioned the implementation of nurse staffing systems in all cases. Second, we describe and explain nurse staffing systems' core components. Third, we analyse the dynamics of these socio‐material relations, the conditioning effects of context and their consequences in two key areas of practice:

a. Unit staffing establishment reviews.
b. Operational risk management.

### 3.1 Implementation Context

The implementation of nurse staffing systems in all cases was mediated by key features of context. First, they were impacted by financial constraints. Budgetary cuts were a factor leading to the failures of care at Stafford Hospital. The Francis Inquiry (Francis 2013) identified that nursing numbers were reduced to save money without adequate accounting for the consequences on patient care or staff morale (Rafferty and Leary 2023). Accordingly, staffing policies in England and Wales were designed to ensure healthcare organisations prioritised safe staffing and care quality. However, following initial investment which led to increases in staff, at the time of the study, staffing establishments in both England and Wales had to be funded from within organisations' budgets, with guidance from England emphasising workforce productivity and efficiency.

Second, systems were conditioned by national workforce shortages. There has been a consistent failure to train enough nurses in the UK (Buchan et al. 2020). The vacancy rate of registered nursing and midwifery staff in Wales was 8.9 at the end of December 2022 (Stats Wales n.d.) and 10.6% within the registered nursing staff group as of June 2023 in England (NHS digital n.d.). There were recruitment challenges in all cases, which included substantive posts and temporary bank and agency staff.

[W]e've got something like two hundred and fifty vacancies […] as quick as we're bringing people in, people are going […] then we're trying to fill the gaps with bank and agency and […] those gaps aren't always filled.

(WCS3/P507/Finance Manager)

Third, implementation was affected by local digital infrastructures. The maturity of systems varied, but no cases had dedicated data analytics facilities and where infrastructures were immature (WCS1, WCS2, WCS3, ECS2, WCS3) the implementation of nurse staffing systems required significant investment of senior nursing resources.

In the true NHS style, we love a system, and we love a database, but what we don't do is invest in the people to manage those systems and databases in a way that gives us really good information.

(ECS2/P404/Senior Nurse)

### 3.2 Core Components

#### 3.2.1 People

The policy intent following the Francis Inquiry, was that safe staffing should be a corporate responsibility, extending from ward to board. Accordingly, in all cases, a wide range of human actors were enrolled in nurse staffing systems: ward‐level nurses; senior nurse managers; general managers; operations, human resources and finance directors and executive board members. Actors were oriented to different operational horizons (ward/unit, service/department, organisation), aligned with different systems of knowledge (clinical, managerial, financial, specialist/generalist), and had different levels of seniority.

#### 3.2.2 Workload Data

All cases deployed formal workload measurement tools. In the English cases, workload was measured used the Safer Nursing Care Tool (SNCT) (Shelford Group n.d.), which is endorsed by the National Institute for Clinical Excellence, a UK public body which provides evidence‐based guidance on health and social care. In Wales, cases deployed the Welsh Levels of Care Tool (WLCT), a national tool, developed through a 2‐year period of ‘iterative testing and refinement’ (NHS Wales 2017). SNCT generates ‘multipliers’, which are used to calculate estimated staffing requirements, whereas WLCT relies on a ‘rigorous process of monitoring staffing levels on a continuous basis’ (NHS Wales 2017). For each staffing establishment review, ward nurses were required to undertake formal retrospective assessments of workload for a determined audit period (minimum of 20 days). Only one case (ECS1) generated real‐time data on workload for operational purposes. Measurement accuracy was an organisational priority. All cases provided extensive training in the use of the workload measurement tools; and three (ECS3, ECS2, WCS2) used external validators to scrutinise the data.

Actor Network Theory (ANT) heightens our awareness of the scripts embedded within formal tools and the assumptions they make about the contexts in which they are applied. Both SNCT and WLCT focus on the patient as the primary unit of measurement. Patients are categorised on a 5‐point scale through a single, composite measure of acuity and dependency. Indirect care, such as organisation and coordination, which makes up a substantial part of registered nursing work (Allen 2015), is omitted from these assessments. Additionally, because measurements are taken at fixed time points, factors like patient turnover, which can significantly impact nursing tasks, are excluded from consideration (Griffiths et al. 2020b). Consequently, both tools tend to underestimate the volume of nurses' workload and provide limited insight into the content of their work. Whilst acknowledging these limitations, clinical nurses valued having access to formal systems of measurement which had been unavailable in the past.

[W]e […] may have three patients in there through the day, who all have an operation, that doesn't seem to be factored in on the tool.

(ECS2/P408/Divisional Director of Nursing)

[W]e had three mental health patients, […] and all require one‐to‐ones, that is not reflected in the Safer Nursing Care Tool.

(ECS2/Fieldnotes/Ward Manager)

So everything we struggle to articulate, and everybody would say in the past, oh, it's anecdote, it's anecdote, it's much easier, because we've got it evidenced.

(ECS1/P114/Nurse Director)

#### 3.2.3 Quality Data

In addition to workload measurement, both systems required staffing decisions to be informed by data on Nurse Sensitive (quality) Indicators (NSI). In Wales, statutory guidance specifies that data should be generated on patient falls, pressure ulcers and medication errors, and ‘any other indicator sensitive to nurse staffing that they deem appropriate’ (Welsh Government 2021). NSI are not prescribed in English policy guidance. In practice, however, all cases depended on three most readily available indicators: pressure ulcers, falls and medication errors. These were drawn from Datix, a national platform on which staff log incidents of harm and can indicate whether poor staffing was a contributory factor. While ease of access is a likely explanatory factor in determining the NSI of choice, it is notable that these are all adverse events. There is no formal standard against which the quality of patient care can be measured, such assessments largely hinge on the professional judgements of nurses. The emphasis on safe, rather than good care, reflects the prevailing emphasis in the research literature on the association of adverse events with nurse staffing levels. Participants questioned whether the NSI deployed in the case studies were sensitive to nurse staffing levels and highlighted the myriad aspects of care quality impacted by staffing shortages, which were not formally measured.

Although we call them nurse sensitive indicators, they're not really aligned substantially just to nursing practice are they?

(WCS2/P312/Nurse Director)

There wasn't that many [pressure ulcers], but that doesn't mean the ward wasn't as safe as it should have been, because there are so many other variables. […] [T]hree patients didn't get washed until three in the afternoon […] we couldn't get to feed the patient at twelve o'clock, and their food was cold. So, there's so much else that is not right, if you haven't got the right staffing levels.

(WCS2/P313/Head of Corporate Nursing)

Generating reliable NSI depended on nurses entering incidents of harm into the Datix platform. Participants indicated that these processes were uneven, with busy clinical staff prioritising patient care rather than data entry.

If you are short‐staffed […] they're more interested in giving care to the patient […] than […] trying to update the information.

(ESC1/P107/Matron)

#### 3.2.4 Professional Judgement

In addition to workload and NSI data, ‘professional judgment’ was an explicit component of both systems. In broad terms, professional judgement refers to assessments of how features of the clinical area impact staffing needs. Both policies refer to the importance of professional judgement in reviewing unit staffing establishments and in making real‐time operational decisions to mitigate risk in response to fluctuating capacity and demand. Wales statutory guidance (Welsh Government 2021) specifies that workforce planning should consider inter alia the experience and skill‐mix of the ward team, the impacts of temporary staff, training and professional development requirements, multiprofessional team dynamics, the impact of ward layout, staff wellbeing, patient turnover and other ward activities, nurses' administrative functions, requirements to support students, and aspects of workload not captured by formal tools. Guidance indicates that professional judgement should be weighted equally with workload and NSI data in workforce planning and, in accounting for staffing decisions, there is a requirement to indicate the prioritisation given to each element. In England, policy refers to ‘clinical’ and ‘managerial’ professional judgement and whilst underlining the importance of combining professional judgment with the SNCT multipliers to determine staffing levels, it does not specify the factors to be considered. Moreover, professional judgement is framed primarily as a mechanism for *interpreting* quantitative data, with guidance explicitly cautioning about the risks of basing decision‐making exclusively on (clinical) professional judgement because of its subjectivity.

Staffing decisions based solely on professional judgement – the expert opinion of clinical staff – are considered subjective and may not be transparent. However professional judgement and scrutiny should be used to interpret the results from evidence‐based tools, taking into account the local context and patient care needs.

(NICE 2014)

In contrast to the priority given to training nurses in systems of measurement, professional judgement was treated as a tacit skill, and in only one case (ECS2) was their formal guidance on its articulation.

### 3.3 Socio‐Material Dynamics in Context I: Unit Staffing Establishment Reviews

Staffing establishment reviews were led by senior nurses and unit managers. Senior nurses were responsible for assembling workload and NSI data, a process described as ‘laborious’.

I have a lot of information about ward establishment […] it takes me like a week. […] That's because everything's on paper and they have loads of different databases. […] and even when they put in data, I know it's all wrong.

(WCS1/P201/Senior Nurse)

Ward managers were central actors for making‐sense of the data, using their knowledge of the care setting to assess whether the metrics aligned with their real‐world experience. These evaluations were oriented to the purposes for which data were used. They were less concerned with accuracy but whether they were a fair representation of the pattern of activity on the unit.

The quality angle is so important. […] if you don't see any incidents. Does that mean that you're providing a quality service? Or is it? It sounds awful, but are you, or haven't you got time to fill in the Datix? […].

(WCS2/301/Senior Nurse)

Those […] who are running those services, are key to first review that data that they've got to say … sense check it. Does it feel right, you know, what's it telling you?

(ECS2/401/Senior Nurse)

Quantitative data on workload and NSI were combined with professional judgement to formulate a provisional staffing plan. The professional judgement component was presented in a narrative format.

It's weighing up everything, […] looking at the ward as a whole […] your staffing levels, […] your acuity, everything else that maybe going on.

[P501: Matron]

Sometimes it's the finer details, and I can only explain by giving clinical scenarios, giving patient experiences, giving staff experiences because […] giving the narrative that surrounds that I think that is important.

(WCS1/P211/Clinical Nurse Director)

In Wales, this triangulated process was recorded in templates which documented the initial establishment plan. Despite its complexity, relatively limited space is allocated for documenting professional judgement. A similar template was deployed in ECS2.

Staffing plans were reviewed at multiple organisational levels in meetings of senior nurses; general managers; and human resources, operations and finance directors, before sign‐off by the Executive Director of Nursing. Here, unit needs were considered in the context of wider organisational concerns and priorities and, in contrast to the contextualised and embedded clinical knowledge of unit managers, decision‐making was dominated by rational‐analytic knowledge systems derived from finance, general management and human resources; knowledge systems detached from the realities of patient care, and which privileged hard data over clinical experience. Not everything that mattered to nurses could be quantified, but despite the acknowledged limitations of workload and NSI metrics, the primacy given to hard data in organisational decision‐making attenuated the authority of nurses' narrative professional judgements, even in Wales where policy required these should be weighted equally with workload and NSI data.

[W]hen you're saying that you need two more nurses because your ward is very busy […] when they ask you, ‘That would be a cost pressure, and what will they actually be doing?’, it's very subjective. So, you're going to say, ‘Well, it's going to improve your patient experience, and it's going to do this and it's going to do that.’ But you can't quantify.

(ECS2/P409/Lead Nurse)

I'm not sure the voice of the nurse around professional judgement is heard. It might be spoken, […] but I'm not convinced it's loud and clear, the pitch from the nurse.

(ECS1/P106/Clinical Lead Nurse)

In addition to the pre‐eminence given to quantitative data in establishment reviews, organisational hierarchies and power relationships conditioned decision‐making. Several participants underlined the challenges clinically‐facing nurses experienced in negotiating with senior colleagues.

[W]ho's going to challenge a band nine divisional director of nursing who says in my professional judgement that's the staffing that we need?

(ECS2/P404/Head of Practice Development)

As a nurse you, you really have to communicate well, because if you don't, it definitely will […] lose the communication further up in at the chain.

(WCS1/P210/Deputy Nurse Director)

Although the UK health service deploys registered nurses and healthcare support workers, neither policy specifies a maximum patient‐to‐nurse ratio or skill‐mix. In all cases, a combination of financial pressures and a challenging recruitment context created an impetus towards skill‐mix dilution. Clinical nurses were asked to think differently about their staffing establishments, to consider whether safe care might be achieved at lower costs, or whether they could work with alternative staffing establishments which there was a more realistic prospect of recruiting to.

[Y]ou could give a ward an extra five qualified nurses, that doesn't mean you're going to get them. […] I think what people are probably doing, […] is knowing that we haven't got that registered nursing workforce out there, is how can we make those areas safe?

(WCS3/P509/Deputy Head of Nursing)

Participants reported a climate in which recruitment was prioritised over workforce planning. In the following extract, a senior nurse reflects on their organisation's success in securing intermediate nursing roles, introduced in England in response to workforce challenges and the lack of a strategy for integrating them into staffing establishments.

The Trust has been amazing at grabbing hold of apprenticeships and new routes into nursing […]. But then when they get here, we don't know what to do with them.

(ECS1/P108/Matron)

In the context of these pressures, nurses struggled to articulate their staffing requirements. Neither workload data, which provided little information on the content of nursing work or the NSI, which could be attributed to staff numbers rather than skills, provided the evidence to inform these decisions.

So, the Board want to know […] why are we going with a Band Seven, why can't it be a Band Six? And that's really difficult. It's easy to verbalise, it's difficult to write, because you know you're writing for an audience that want data, more than narrative, but the narrative is that expert knowledge, around why you need that staffing?

(WCS2/P313/Head of Corporate Nursing)

Ward managers were required to sign off their final establishment plans, and respondents indicated that formal disagreements were rare. Nevertheless, our data suggest that this may reflect the power dynamics of organisational decision‐making, rather than providing evidence of ward‐level consensus.

I bet no one is going to tell me that I'm going to get extra nurse because of my acuity. Everyone will tell me that your staffing is staying the same because we haven't got money. So, I don't really see the point […] when nothing changes.

(ECS1/P105/Matron)

So, we've had to reduce the skill‐mix, in that way, there will be consequences, however well you're managing it. There will be consequences for sure, patients not getting the level of observation and supervision, they would have otherwise.

(ECS3/603/Managing Director)

### 3.4 Socio‐Material Dynamics in Context II: Managing Operational Risk

Unit establishment‐setting determined the average staffing level required for normal operations, but healthcare systems are inherently turbulent. Patients within the same classification can have different care needs, admissions and discharges are unpredictable, staff of the same grade may have different skills and experience, and rosters impacted by unplanned absences and vacancies. National policies required organisations to respond to these dynamic fluctuations.

In all cases, as a result of wider workforce and recruitment challenges, there was rarely sufficient staff to fill planned rosters.

The challenge plain and simple is with all the will in the world and with all the money in the world, we are finding it difficult to meet the planned rosters.

(WCS3/P502/Clinical Nurse Director)

All sites had formal escalation policies for mitigating risks if there was a gap between planned and required staffing levels. These included guidance on ward‐level interventions, through to operational decisions to close services. While establishment‐setting involved a wide range of organisational actors, it was primarily nurses who bore responsibility for ensuring safe staffing for operational purposes. Moreover, although quantitative data played a key role in establishment‐setting, effective operational risk management relied almost exclusively on the professional judgments of nurses.

But ultimately, irrespective of what the numbers are telling you, the professional judgement probably will override that, because sometimes there's nothing you can do about the numbers.

(ECS1/P108/Matron)

Risk was an organisational issue. Senior nurses were responsible for balancing clinical nurses' concerns for their areas with the management of risk across the organisation.

It's not about having one area that is absolutely okay and then everything else is crumbling.

(WCS2/P314/Senior Nurse Patient Flow)

[W]hat's the best I can do with the staff that I've got? […] How do I equalise the risk across the organisation?

(ECS3/P603/Managing Director)

They continuously monitored overall capacity and demand, through multiple meetings, the use of available technologies, visits to the wards, and on‐going dialogue with colleagues. In ECS1, the digital system enabled ward‐level data on workload and staffing levels to be reviewed centrally and shared with senior nurses. But elsewhere the digital infrastructures were more limited, and maintaining an organisational overview placed heavy burdens on senior nurses, who were required regularly to visit the wards in their areas.

I can't go on a computer anywhere in the health board and see what the ward staffing is. I have to go to a ward, when I'm on call, with a pen and paper, […] that starts at seven, and if I'm lucky, by 9.30, I'll have got around all the wards.

(WCS2/P201: Senior Nurse)

In all cases, there was an expectation that ward nurses should act to mitigate risk in their areas. This included adjusting the roster, redeploying staff (specialist nurses, practice educators), asking allied health professionals to help, drawing staff into the establishment that were supposed to be supernumerary (ward managers, student nurses), organising work differently, ‘cohorting’ patients at risk of falls in a single area, modifying patient care plans (e.g. asking medical staff to change medications from intravenous to oral), negotiating with another ward to cover short‐term demands, and deferring new admissions or ward transfers. There were clear organisational pressures for units to manage within existing capacity rather than requesting additional staff, with senior nurses central in supporting these decisions.

Now part of the work that we've been doing is to get people, rather than saying I need an extra nurse, is to think about how they're working, and maybe start to swap around who, who keeps that close observation of that patient.

(ECS3/P601/Associate Nurse Director)

[S]omebody rings you up and says, ‘Ooh, it's not safe […] I want another nurse.’ ‘Well, I haven't got one, so we'll close the unit.’ That always makes people really think how safe it is. […] So, you will try that first, to get people to understand what they're asking you, and what they can cope with.

(ECS2/P408/Divisional Nurse)

In several cases there had been deliberate strategies to enrol ward nurses into the wider organisational agenda. In WCS1 and WCS2 ward managers attended staffing meetings in which data for the whole organisation was shared. In other cases (ECS2, ECS2), staffing data was widely available at ward level. The intent was to encourage an organisational‐level view and discourage protectionist practices.

[T]hey can look at staffing on their own areas, and across other areas. […] so they have an understanding of the wider issues, as well as within their own ward area.

(ECS3/P604/Matron)

The whole thing for me is around getting the ward managers to […] understand what is happening in other areas compared to what's happening in their area and how we mitigate risk across the system.

(WCS2/P314/Senior Nurse Patient Flow)

Staff redeployment was the most common strategy to mitigate risk. While formal escalation plans in all cases included closing clinical areas, our data include no examples of such events.

I expect my staff to be moved, I expect not to be fully staffed, I expect there to be shortages throughout the day and I suppose we're all trying to work with that as best we can because we know that there's nowhere to turn to for more staff.

(ECS3/P609/Ward Manager)

The constant movement of staff had negative impacts on wellbeing and morale, with attendant implications for staff retention.

We had two wards […] they had great staff retention, they were well managed, they were well led, brilliant wards, always had full staff. Other areas were short every single day, so it got to a point where we were pulling nurses from these good wards, every single day, and the knock‐on effect is the staff then got fed up, […] and […] started to leave. […] every day they were coming to work not knowing where they were going to end up.

(ECS1/P109/Director of Improvement)

Ward managers are not included in the staffing establishment in England and Wales, reflecting the importance of their clinical leadership role, but in the context of staff shortages, they often took a clinical caseload.

I would put myself in the numbers, but then […] next day I come to work and I have like 80 emails to respond, or to look after operational side or something.

(ECS1/P105/Matron)

For senior nurses, risk‐mitigation was a heavy psychological burden. This reflected the inherent uncertainty in the process and the impact of decision‐making on relationships with clinically‐facing colleagues.

So, it is an unhappy role, […] the senior nurse who is making those decisions, because you are quite unpopular. They have to have enough professional weight to be able to make those decisions and stand behind them, because, on ward a) where you're moving the staff, you may have people say, ‘Oh that's completely unsafe, it's dangerous’, but they've got to take that judgement. […] They have to have big enough boots to make decisions and to make the right decision for everybody, which is often the tough one.

(ECS3/P603/Managing Director)

In the clinical areas, there was evidence of extensive professional disquiet about care quality.

You will describe a total shitshow to them and then the matron will say ‘but it's okay we have a plan to mitigate that’.

(ECS2/Fieldnotes)

[W]e have to make it safe, but […] people don't want it to be safe, they want it to be good, they want it to be better than safe.

(ECS3/P609/Ward Manager)

While formal systems were in place in England and Wales to enable staff to ‘speak up’ if they had professional concerns about care quality, there were no references in our data to nurses formally raising concerns. These findings are indicative of moral injury, which risks perpetuating existing workforce challenges and/or normalising poor quality care.

## 4 Discussion

This paper has examined the socio‐material relations involved in accomplishing ‘safe’ nurse staffing in six case studies in England and Wales. It has highlighted the impacts of financial constraint, workforce shortages and immature digital infrastructures on system implementation; the limitations of formal measurement systems for informing difficult workforce planning decisions or monitoring their effects; and how strategic and operational practice contexts mediated the distribution of decision‐making agency between human and nonhuman actors. Nurse staffing systems consumed significant senior nurse leadership time and expertise, but despite policy intent, all cases reported a drift towards skill‐mix dilution and the routine requirement to deliver services with insufficient staff. The attendant impacts on care quality and staff wellbeing, created system spiralling effects which negatively impacted pressurised services.

Healthcare systems and nursing face a challenging future. Discontent about staffing levels is affecting workforce retention, the attractiveness of nursing as a career, and student nurse attrition. Governments across the world are implementing various strategies to bridge the gap between capacity and demand. This study has illustrated how a socio‐material approach has value for better understanding nurse staffing systems and raises several broader issues for consideration to strengthen their effectiveness in the diverse contexts of their use.

Quantitative data is a powerful actor in nurse staffing systems, as it furnishes the evidence required in the rational‐analytic contexts of organisational decision‐making and facilitates transparency and accountability. But, as we have previously argued, our findings raise important questions about what we quantify, how, and with what effects (Allen et al. 2023). While most nurse staffing systems include consideration of NSI, selecting appropriate indicators is challenging. Given there is no evidence of optimal staffing levels (Griffiths et al. 2020a) and nurse‐patient ratios vary internationally within different staffing policies (Van den Heede et al. 2020), practising safe staffing is inextricably linked to the more challenging question about the standard of patient care healthcare systems are prepared to pay for and how this can be measured (Allen et al. 2023). In this context, identification of appropriate NSI is an important priority. Blume et al. (2021) assessed the strength of the evidence for the association of staffing levels with 22 NSI. The indicators used in our case studies had only low (pressure ulcers, falls) and moderate levels of evidence (medication errors), but length of stay, readmissions and patient satisfaction are strongly associated with nurse staffing and merit closer consideration. As measures which are routinely generated by healthcare organisations, their use would also reduce data generation burdens for clinical staff and strengthen alignment of clinical and corporate interests in underlining the nursing contribution to organisational efficiency, in a context in which nursing is consistently framed as an organisational cost, rather than an investment.

Nursing workload can be measured in diverse ways and systems need to be aligned with the workforce planning requirements of the practice context. Patient classification and time‐task measures may have value in healthcare systems with a predominance of registrants, but where there is a mixture of registered and licensed nurses and support workers, more granular measurement systems are necessary to inform decision‐making. Healthcare systems are evolving internationally in response to the legacy effects of the COVID‐19 pandemic, demographic changes, and the promise of new technologies. In England and Wales, new intermediate nursing associate roles are emerging, signalling an increasingly complex skills‐mix; there are plans to shift care away from acute hospital settings, with impacts for community nursing; and in the future, new technologies are likely to impact the distribution and organisation of care work (NHS England 2023; Welsh Government 2023). In such contexts, more sophisticated systems are necessary to measure the full range of direct and indirect nursing and care work, as well as any workload impacts arising from wider skill‐mix changes, new technologies or changes to work content.

Whether implicit or explicit, professional judgement is a necessary component of staffing systems because of the complex ways in which clinical environments effect workforce requirements and the fallibility of any system of measurement. As our case studies illustrate, however, healthcare organisations are characterised by epistemic and status hierarchies, and the authority of professional judgement cannot be taken for granted. Since the cessation of our research, Saville et al. (2023) have published a framework designed to support nurses in England to deploy their professional judgement in staffing establishment reviews. This is important progress, but more could be done. Alongside growing evidence of the value of clinicians in the governance of healthcare organisations (Kirkpatrick et al. 2023), other studies have highlighted the challenges of clinical voices being crowded out by managerial perspectives and/or the risk that clinical leaders may come to share the corporate interests of managers (Waring and Bishop 2013).

Clinical environments are complex dynamic systems. On the one hand, there is a need to better prepare nurses to articulate this understanding to nonclinical audiences, through undergraduate preparation and professional development programmes. On the other hand, there is a need to improve organisational understanding and recognition of nursing expertise. It is noteworthy perhaps, that whilst there were extensive efforts in some of our cases to enrol nurses in organisational agenda, there were no reciprocal initiatives to improve corporate understanding of the complexity of front‐line service delivery. Professional judgement is typically treated as tacit knowledge (Polanyi 1958), but no knowledge is intrinsically tacit (Wyatt 2001). Consideration should also be given to the development of structured reporting templates which evidence those elements of a clinical setting that impact staffing need, aligned with digital systems which generate the data necessary to inform and evidence those judgements. These have potential to function as ‘boundary objects’ (Star and Griesemer 1989) to facilitate conversations between clinical and corporate actors in developing strategic workforce plans.

Many nurse staffing systems have required significant investment in development and training, with several embedded in proprietary digital management infrastructures. Given the limited evidence on which to recommend any specific tool or methodology the priority for future research is to identify how existing systems might be refined and their costs and benefits (Griffiths et al. 2020a). To better understand the relationships between the core components of nurse staffing systems, and how these are conditioned by implementation contexts, future research would benefit from study designs that combine a socio‐material perspective with realist evaluation paradigm (Pawson and Tilley 1997). Realist evaluation entails the development and testing of theories about the relationships between context, mechanisms and outcomes, and has recently been extended to include consideration of costs and resource utilisation (Anderson and Hardwick 2016). ANT and practice‐based approaches have value in informing both initial theory development and subsequent data generation and analysis. In research on specific staffing systems, studies of this kind could address the question of what works, for whom, in what respects and at what cost. Furthermore, in opening the black box of staffing systems, they also have the potential to generate transferrable insights for improving other models, including modification of resource‐intensive elements that do not add value.

## 5 Conclusion

Nursing is at a critical juncture. Amid rising concerns over healthcare quality, escalating costs, workforce shortages, and the rapid advancement of new technologies, fundamental questions about the ideal number of registered nurses required to meet consistent care standards remain unresolved (Griffiths et al. 2020a; Allen et al. 2023). In this context, methods for determining appropriate nurse staffing levels must be robust and adaptable. Adopting a socio‐material, practice‐based approach offers valuable insights for understanding and enhancing existing systems as healthcare continues to evolve in response to the complex demands of late modernity.

## Ethics Statement

The study received ethics approval from Cardiff University School of Healthcare Sciences Research Ethics Committee, and all participants gave full written consent.

## Conflicts of Interest

The authors declare no conflicts of interest.

## Data Availability

The authors have nothing to report.
